# Curcumin and piperine supplementation of obese mice under caloric restriction modulates body fat and interleukin-1β

**DOI:** 10.1186/s12986-018-0250-6

**Published:** 2018-02-06

**Authors:** Taiki Miyazawa, Kiyotaka Nakagawa, Sharon H. Kim, Michael J. Thomas, Ligi Paul, Jean-Marc Zingg, Gregory G. Dolnikowski, Susan B. Roberts, Fumiko Kimura, Teruo Miyazawa, Angelo Azzi, Mohsen Meydani

**Affiliations:** 1 Vascular Biology Laboratory, Jean Mayer USDA-Human Nutrition Research Center on Aging, (HNRCA) at Tufts University, Boston, MA 02111 USA; 2 Food and Biodynamic Chemistry Laboratory, Graduate School of Agricultural Science, Tohoku University, Sendai, 980-0845 Japan; 3 Vitamin Metabolism Laboratory, Jean Mayer USDA-Human Nutrition Research Center on Aging, (HNRCA) at Tufts University, Boston, MA 02111 USA; 4 Department of Biochemistry and Molecular Biology, Miller School of Medicine, University of Miami, Miami, Florida, 33136 USA; 5 Mass Spectrometry Laboratory, Jean Mayer USDA-Human Nutrition Research Center on Aging, (HNRCA) at Tufts University, Boston, MA 02111 USA; 6 Energy Metabolism Laboratory, Jean Mayer USDA-Human Nutrition Research Center on Aging, (HNRCA) at Tufts University, Boston, MA 02111 USA; 7 Food and Biotechnology Innovation Project, New Industry Creation Hatchery Center (NICHe), Tohoku University, Sendai, 980-8579 Japan; 8 Food and Health Science Research Unit, Graduate School of Agricultural Science, Tohoku University, Sendai, 980-0845 Japan

**Keywords:** Caloric restriction, Curcumin, Glucuronide, High fat diet, Inflammation, Meso scale discovery, Metabolic syndrome, Obesity, Piperine, Tandem mass spectrometry

## Abstract

**Background:**

Dietary bioactive compounds capable of improving metabolic profiles would be of great value, especially for overweight individuals undergoing a caloric restriction (CR) regimen. Curcumin (Cur), a possible anti-obesity compound, and piperine (Pip), a plausible enhancer of Cur’s bioavailability and efficacy, may be candidate agents for controlling body fat, metabolism and low grade inflammation.

**Methods:**

47 eight-week-old male C57BL/6 mice were fed a high fat diet (HFD) for 23 weeks to induce obesity. Then, mice were divided into 5 groups. Group 1 continued on HFD ad libitum. The other 4 groups underwent CR (reduced 10% HFD intake for 10 weeks, 20% for 20 weeks) with Cur, Pip, Cur + Pip or none of these. Percent body fat, plasma inflammatory markers associated with obesity (interferon (IFN)-γ, interleukin (IL)-10, IL-12 p70, IL-1β, IL-6 and KC/GRO), plasma Cur metabolites and liver telomere length were measured.

**Results:**

Compared to the other groups, obese mice who underwent CR and received Cur + Pip in their diet lost more fat and had significantly lower IL-1β and KC/GRO. Tandem mass spectrometry analysis of plasma from obese mice under CR showed no difference in Cur metabolite levels between groups supplemented with Cur alone or combined with Pip. However, plasma IL-1β levels were inversely correlated with curcumin glucuronide. Minor modulation of telomere length were observed.

**Conclusions:**

It is plausible that supplementing the high fat diet of CR mice with Cur + Pip may increase loss of body fat and suppresses HFD induced inflammation. Combination of Cur and Pip has potential to enhance CR effects for the prevention of metabolic syndrome.

## Background

**Fig. 1:**
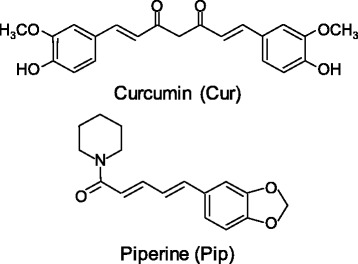
Chemical structures of spice compounds, curcumin (Cur) and piperine (Pip)

Recent studies show that caloric restriction (CR) modulates energy expenditure and body fat metabolism, it also regulates telomere length, which is associated with longevity in some experimental animals [1–3]. Other studies suggest that certain dietary components found in spices may exert a beneficial effect on metabolism. For example, curcumin (Cur, Fig. 1), the major polyphenol present in the spice turmeric, possess anti-obesity and anti-inflammatory properties [4–6]. Piperine (Pip, Fig. 1), a bioactive alkaloid in pepper, has been suggested to increase Cur’s absorption and bioavailability [7]. In addition, Cur and Pip have been suggested to attenuate inflammation [8, 9]. Our previous study sought to evaluate whether Cur and/or Pip could potentiate CR’s effect on body weight reduction in mice made obese by feeding a high-fat diet (HFD) [10]. CR favorably impacted metabolic profiles of obesity (i.e., lower body weight, plasma glucose and plasma insulin) in obese mice, but the addition of Cur and/or Pip into the CR diet resulted in no further measurable benefit on total body weight.

Telomeres are important component of cell division that protect the genome DNA against cellular senescence and promote chromosomal stability. Shortening of the telomeres and DNA damage is associated with age-related disorders, obesity, immune dysregulation, cardiovascular disease and cancer [11–13]. Several studies reported an inverse association of telomere length with obesity [3, 14, 15]. The effect of Cur and/ or Pip on telomere length in obese mice undergoing CR is not known. In the present study, we further investigated whether Cur and/or Pip could improve metabolic profiles in obese mice subjected to CR on a HFD. To this end, we measured the changes in body weight, area under the curve (AUC) of percent total body fat, telomere length and several markers of inflammation.

## Methods

### Animals, diets, and treatments

**Fig. 2:**
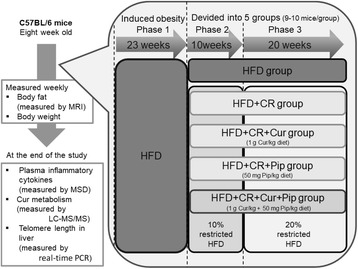
Experimental design of the present study. CR, Caloric restriction; Cur, Curcumin; HFD, High-fat diet; HPLC-MS/MS, Chromatography with tandem mass spectrometry; MRI, Magnetic resonance imaging; MSD, Meso scale discovery; Pip, Piperine

Our goal was to evaluate whether the beneficial effect of CR on reduction of HFD-induced obesity could be further potentiated by concurrent supplementation with dietary Cur, with or without the addition of Pip. The experiment of feeding was designed to mimic a situation in which obese individuals select a long term [5, 10], CR regimen to lose body weight (mainly body fat), possibly accelerating the loss of body weight by combining the CR regimen with intake of certain dietary bioactive compounds or drugs known to upregulate basal metabolism and further accelerate weight loss. Thus, the study protocol consisted of three phases (Fig. 2). During the 23 weeks period of Phase 1, all 47 eight-week-old male C57BL/6 mice (Jackson Laboratory, ME) were fed a Western style HFD (Harlan Teklad, WI, formulation #TD 06433, 44% calorie from fat) to induce obesity. Mice were then divided into 5 groups (9–10 mice/group). Group 1 (*n* = 9) continued consuming HFD ad libitum for the rest of study (30 weeks). This group of mice was included in the study design to demonstrate maximum body weight gain and percent of adiposity. The remaining 4 groups underwent CR, first by 10% for 10 weeks (Phase 2), then by 20% (Phase 3) for 20 weeks through the rest of the study. For 10 and 20% calorie restricted, we fed the mouse with 10 or 20% of less food. 10 or 20% food was determined based on consumption of one week consumed by each individual during the Phase 1. During Phase 2 and 3, the HFD of Group 3 was supplemented with 1 g Cur/kg diet, the HFD of Group 4 was supplemented with 50 mg Pip/kg diet, and the HFD of Group 5 was supplemented with 1 g Cur/kg + 50 mg Pip/kg diet. Mice were weighed twice a week, and starting from week 23, their body fat was measured once every two weeks by a small animal magnetic resonance imaging (MRI) system (EcoMRI, Ecomedical System, TX). To further elaborate on the effect of CR and Cur and/or Pip on the percent of body fat loss, the area under the curve (AUC) of body fat were plotted and AUCs were calculated using Image J (NIH, NY). Numeric results were tabulated in Excel files for statistical analyses.

The mice were housed individually in shoebox polycarbonate cages under 12 h light/dark cycles. Water was provided ad libitum*.* At the end of the study, mice were killed by CO_2_ asphyxiation followed by exsanguination. Blood was collected by cardiac puncture into EDTA-containing tubes. Then, plasma was separated from red blood cells and buffy coat by centrifugation at 1000 *g* for 10 min at 4 °C and stored at − 80 °C until analysis. Liver was collected and stored at − 80 °C until analysis.

### Inflammatory cytokines

Obesity is associated with a chronic, low grade inflammation which potentially contributes to the increased risks of metabolic syndrome [16, 17]. Therefore, we sought to determine if supplementing the HFD of CR mice with Cur, with and without Pip, would reduce the expression of inflammatory cytokines. Plasma samples were analyzed for a battery of cytokines including interferon (IFN)-γ, interleukin (IL)-10, IL-12 p70, IL-1β, IL-6, Keratinocyte chemoattractant / growth-regulated oncogene chemokines (KC/GRO, IL-8 related protein) using a multiplex kit by Meso Scale Discovery (MSD, MD) according to manufacturer’s instructions.

### Cur metabolites

It has been reported that Pip can increase Cur bioavailability, but it is not known if this effect is a direct effect of Pip on Cur metabolism [7]. Thus, using a liquid chromatography with tandem mass spectrometry (HPLC-MS/MS) system, we analyzed plasma samples from the mice treated with Cur or both Cur and Pip (Cur + Pip) [18]. Briefly, plasma (200 μL) was mixed with 400 μL of methanol by vortex and centrifuged at 5000 *g* for 15 min at 4 °C. The supernatant was collected, mixed with 1 mL of water, and loaded onto an Oasis HLB 1 cm^3^ cartridge (Waters, MA). The cartridge was washed with 1 mL water, and curcuminoids (Cur and metabolites) were eluted with 2 mL methanol. An aliquot of the extract was injected into a C18 column (XBridge C18, 2.1 × 150 mm, Waters, MA) kept at 40 °C. The mobile phase consisted of two components: A) 0.05% formic acid (pH 3.0) and B) acetonitrile. The gradient profile was as follows: 0–30 min, 85–0% A linear. The flow rate was 0.2 mL/min. Curcuminoids were analyzed using API 3200 QTRAP HPLC-MS/MS (AB SCIEX, CA). Electrospray negative ionization was used as an ion source with collision energy of − 60 V, declustering potential of − 45 V, turbo gas temperature at 300 °C, and spray voltage of − 4500 V. Other parameters were: nebulizer gas, 30 psi; auxiliary gas, 30 psi; curtain gas, 20 psi; collision gas, medium. Curcuminoids were detected using multiple reaction monitoring (MRM) for the transition of parent ions to product ions: Cur, *m/z* 367 > 134; curcumin glucuronide (CurG), *m/z* 543 > 134; curcumin sulfate (CurS), *m/z* 447 > 134; curcumin glucuronide sulfate (CurGS), *m/z* 623 > 134; curcumin diglucuronide (CurDG), *m/z* 719 > 134; curcumin disulfate (CurDS), *m/z* 527 > 134; dihydrocurcumin (DHCur), *m/z* 369 > 135; tetrahydrocurcumin (THCur), *m/z* 371 > 135; hexahydrocurcumin (HHCur), *m/z* 373 > 179; octahydrocurcumin (OHCur), *m/z* 375 > 179; dihydrocurcumin glucuronide (DHCurG), *m/z* 545 > 135; tetrahydrocurcumin glucuronide (THCurG), *m/z* 547 > 135; hexahydrocurcumin glucuronide (HHCurG), *m/z* 549 > 179; octahydrocurcumin glucuronide (OHCurG), *m/z* 551 > 179; dihydrocurcumin sulfate (DHCurS), *m/z* 449 > 135; tetrahydrocurcumin sulfate (THCurS), *m/z* 451 > 135; hexahydrocurcumin sulfate (HHCurS), *m/z* 453 > 179; octahydrocurcumin sulfate (OHCurS), *m/z* 455 > 179 [19]. Concentrations of CurG in plasma were calculated using the standard curve of CurG.

### Telomere length

It is believed that some of CR’s health benefits are associated with the prevention of telomere shortening [3]. We sought to determine if CR imposed on HFD provides the same protection as CR imposed on a normal mouse diet. Thus, we measured the telomere lengths in liver samples of all 5 groups of mice. DNA from liver tissue was isolated using DNeasy blood and tissue kit (Qiagen, CA). Telomere length was determined using a real-time PCR based assay described by Cawthon et al. [20], which was adapted for mice by Kotrschal et al. [21]. In this assay, telomeric repeats were amplified with primers specific for the region, and a single copy gene was amplified as control for input DNA. The relative telomere length was expressed as the ratio of telomere repeat to single copy gene (T:S ratio). The PCR reactions were carried out using Power SYBR Green PCR master mix (Life Technologies, CA) and Applied Biosystems 7300 Real-Time PCR system (Life Technologies, CA). A standard curve prepared from mouse genomic DNA (Promega, WI) was present in every plate and was used to quantitate the telomere or single copy gene in the samples.

### Statistical analysis

The nonparametric Kruskal-Wallis test, followed by Dunn’s multiple comparison test, was performed using Prism 6 for Windows Version 6.01 (GraphPad Software, CA). Differences were considered significant at *P* < 0.05. For correlation analysis, Spearman’s rank correlation coefficient test was used.

## Results

### Body weight and body fat

**Fig. 3:**
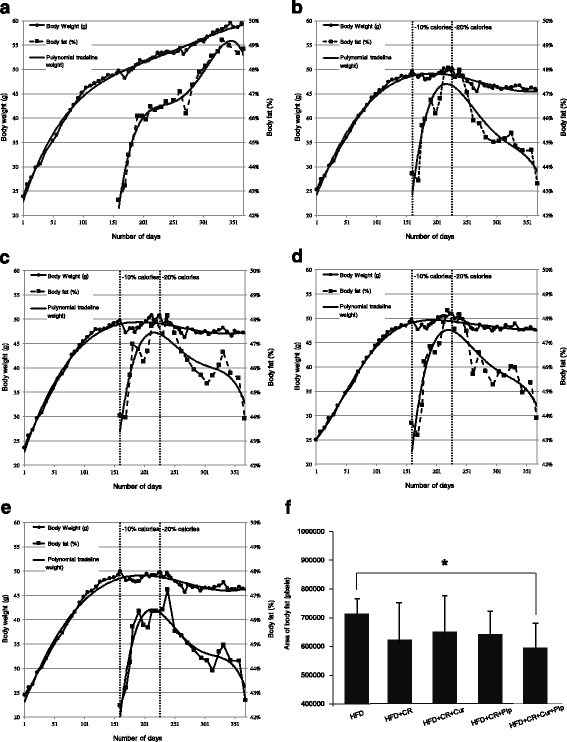
**a-e** Body weight and % of total body fat in each group. Body weight measured once a week, percent adiposity was measured by MRI every two weeks. **a** Group 1 high-fat diet (HFD), **b** Group 2 (HFD+ caloric restriction (CR)), **c** Group 3 (HFD + CR+ curcumin (Cur)), **d** Group 4 (HFD + CR+ piperine (Pip)), **e** Group 5 (HFD + CR + Cur + Pip). Data were expressed as mean values. **f** Comparison of total percent body fat (area under the curve (AUC)) of each groups measured during the phase 3 (20% CR, *n* = 9–10). *P < 0.05 compared to Group 1 by Dunn’s multiple comparisons test. Data were expressed as mean ± standard deviations (SD)

The percent of total body fat of each mouse group was plotted and presented in Fig. 3. During Phase 1, which was 23 weeks, all mice were fed a HFD ad libitum. Group 1 continued eating HFD ad libitum to the end of the study. Over the 53 weeks of the study, mice gained significant body weight (~ 60 g) with as much as 49% of their weight consisting of total body fat. During Phase 2, when total caloric intake was reduced by 10% CR, body weight gain was stabilized and remained unchanged, but the percent of total body fat as measured by MRI increased substantially (Fig. 3). However, while they were consuming HFD at 20% CR (Phase 3), the loss of total body weight was small, but the percent of body fat loss was significant over the 20 weeks period. As shown in Fig. 3f, supplementing the CR diet of mice with Cur + Pip over the 20 weeks period significantly (*P* < 0.05) reduced AUC of percent adiposity compared to ad libitum fed animals. Supplementing CR diets with Cur or Pip alone was not effective at increasing further body fat loss.

### Plasma inflammatory cytokines

**Fig. 4:**
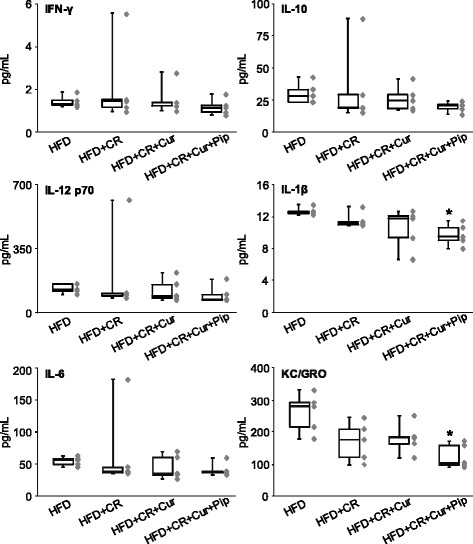
Effect of CR and bioactive components of spice on plasma inflammatory cytokines. Plasma interferon (IFN)-γ, interleukin (IL)-10, IL-12 p70, IL-1β, IL-6 and keratinocyte chemoattractant / human growth-regulated oncogene chemokines (KC/GRO) were measured by multiplex kit by meso scale discovery (MSD). Detailed analytical conditions are described in the materials and methods section. Each box plot shows the maximum (top of the vertical line), 75th percentile (top of the box), median (middle line in the box), 25th percentile (bottom of the box), and minimum (bottom of vertical line) values of data (*n* = 5). *P < 0.05 represents significant differences between Group 1 high-fat diet (HFD) and Group 5 (HFD + caloric restriction (CR) + curcumin (Cur) + piperine (Pip)) by Dunn’s multiple comparisons test

Mice treated with Cur + Pip had significantly lower levels of cytokines IL-1β and KC/GRO compared to the mice fed HFD ad libitum (*P* < 0.05, Fig. 4). We did not observe any effect of Cur and Cur + Pip on other inflammatory parameters (IFN-γ, IL-10, IL-12 p70 and IL-6). These results indicate that Pip may contribute to Cur reduction of inflammatory processes by decreasing the levels of IL-1β and KC/GRO through suppression of adipose tissue inflammation. IL-1β is an inflammatory cytokine that is produced by activated macrophages, and stimulate release of inflammatory mediators such as prostaglandin [22]. KC/GRO is a potent neutrophilic chemotactic cytokine that participate in recruitment of neutrophil in acute mast cell-dependent skin inflammation [23]. These cytokines would be attenuated by the anti-inflammatory activity of Cur or Pip individually with the administration of optimal concentrations, or possibly through combined anti-inflammatory activities of both Cur and Pip.

### HPLC-MS/MS evaluation of cur metabolism

**Fig. 5:**
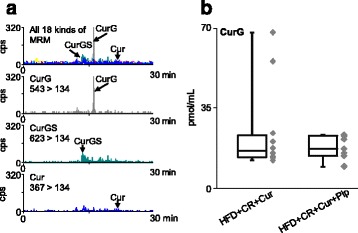
Chromatography with tandem mass spectrometry (HPLC-MS/MS) analysis of curcumin (Cur) metabolism. **a** Plasma curcuminoids were analyzed by HPLC-MS/MS with 18 kinds of MRM (Cur, *m/z* 367 > 134; curcumin glucuronide (CurG), *m/z* 543 > 134; curcumin sulfate (CurS), *m/z* 447 > 134; curcumin glucuronide sulfate (CurGS), *m/z* 623 > 134; curcumin diglucuronide (CurDG), *m/z* 719 > 134; curcumin disulfate (CurDS), *m/z* 527 > 134; dihydrocurcumin (DHCur), *m/z* 369 > 135; tetrahydrocurcumin (THCur), *m/z* 371 > 135; hexahydrocurcumin (HHCur), *m/z* 373 > 179; octahydrocurcumin (OHCur), *m/z* 375 > 179; dihydrocurcumin glucuronide (DHCurG), *m/z* 545 > 135; tetrahydrocurcumin glucuronide (THCurG), *m/z* 547 > 135; hexahydrocurcumin glucuronide (HHCurG), *m/z* 549 > 179; octahydrocurcumin glucuronide (OHCurG), *m/z* 551 > 179; dihydrocurcumin sulfate (DHCurS), *m/z* 449 > 135; tetrahydrocurcumin sulfate (THCurS), *m/z* 451 > 135; hexahydrocurcumin sulfate (HHCurS), *m/z* 453 > 179; octahydrocurcumin sulfate (OHCurS), *m/z* 455 > 179). Typical chromatograms for all MRM combined and three MRM representatives (CurG, CurGS, and Cur) were shown. Detailed analytical conditions are described in the materials and methods section. **b** Plasma CurG concentrations of high-fat diet (HFD) + caloric restriction (CR) + Cur and HFD + CR + Cur + piperine (Pip) groups were plotted. Each box plot shows the maximum (top of the vertical line), 75th percentile (top of the box), median (middle line in the box), 25th percentile (bottom of the box), and minimum (bottom of vertical line) values of data (*n* = 9–10)

Since supplementation of CR obese mice with Cur + Pip significantly lowered the plasma levels of IL-1β and KC/GRO (Fig. 4, *P* < 0.05), we performed HPLC-MS/MS analysis to evaluate whether this phenomenon depends on the alteration of Cur metabolism by Pip. The analysis revealed that, in CR obese mice, most of the Cur existed in a glucuronide-conjugated form (CurG) (Fig. 5a). Other conjugates (CurS, CurDG, CurDS and CurGS) and Cur were found in plasma at extremely low levels. There were no detectable levels of reductive metabolites (DHCur, THCur, HHCur, OHCur, DHCurG, THCurG, HHCurG, OHCurG, DHCurS, THCurS, HHCurS and OHCurS). No significant differences were observed in CurG levels between groups supplemented with Cur or Cur + Pip (Fig. 5b), thus we can infer that the lower levels of cytokines (IL-1β and KC/GRO, Fig. 4) were not due to Pip’s effect on Cur metabolism. Finally, we plotted plasma CurG concentrations versus other parameters (IFN-γ, IL-10, IL-12 p70, IL-1β, IL-6 and KC/GRO) measured in the Cur + Pip supplemented groups. There was a significant negative correlation between plasma CurG and IL-1β (*r* = − 0.90, *P* = 0.002, Table 1). This correlation was not present between CurG and other markers (IFN-γ, IL-10, IL-12 p70, IL-6 and KC/GRO). Hence, there might be some possibility that CurG acts independently as an anti-inflammatory agent to reduce plasma IL-1β.

**Table 1:** Correlations between CurG and plasma paramaters in group 5 (HFD + CR + Cur + Pip)

Parameters	r (n = 9–10)	*P*
Glucose	0.28	0.305
Insulin	0.12	0.661
IFN-γ	−0.40	0.320
IL-10	−0.31	0.456
IL-12	−0.62	0.102
IL-1β	−0.90	0.002
IL-6	−0.60	0.120
KC/GRO	−0.12	0.779

Analyzed by Spearman’s rank correlation coefficient test

*CurG* curcumin glucuronide, *KC/GRO* keratinocyte chemoattractant / human growth-regulated oncogene chemokines, *IFN* interferon, *IL* interleukin

### Telomere length in liver

**Fig. 6:**
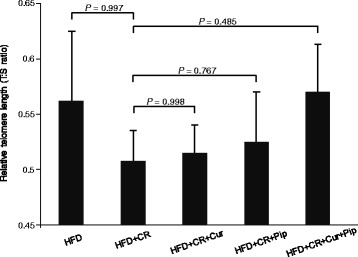
Liver telomere length measured using a real-time PCR based assay. The relative telomere length was expressed as the ratio of telomere repeat:single copy gene (T:S ratio). A standard curve prepared from mouse genomic DNA (Promega, WI) was present in every plate and was used to quantitate the telomere or single copy gene in the samples (n = 9–10). Data were expressed as mean ± standard error of the mean (SEM). *P* values between each groups were confirmed by Dunn’s multiple comparisons test

Cur has antioxidant and anti-inflammatory properties, which may protect against telomere shortening [24]. However, the impact of Cur and/or Pip on telomere length in CR mice fed HFD is not clear. Therefore, we measured telomere length in the liver of obese mice under CR (Fig. 6). In contrast to our expectation that CR would prevent telomere shortening, we found that CR in HFD-supplemented mice led to a marked reduction of telomere length. However, the negative effect of combined CR and HFD on telomere shortening was modulated by Cur + Pip supplementation (*P* = 0.485, Fig. 6). These results may indicate that when HFD regimen is subjected to CR immediately, telomere length is probably affected by oxidative stress. However, the inclusion of Cur or Pip into the diet suppressed telomere shortening to some extent. This effect was further enhanced when Cur + Pip was included in the diet.

## Discussion

CR, known to modulate energy balance, is an effective way to reduce the risk of metabolic syndrome and obesity in humans [1, 2], and great interest exists to accelerate this process in order to reach an ideal body weight. To this end, Cur has been shown to have the potential to prevent chronic inflammatory diseases and has recently emerged as a food component with the potential to suppress body weight gain [4]. In our earlier studies, we demonstrated that Cur may have anti-obesity properties [5]. However, its low bioavailability appears to be a hindrance to its health benefits. To overcome this drawback, Pip has been shown to increase absorption of Cur from the GI tract and increase its bioavailability [7]. We found that during Phase 2 of our study (10% CR), the average bodyweight of mice stabilized at approximately 50 g. At the end of Phase 3 (20% CR), the average bodyweight had dropped to nearly 40–45 g. Supplementing HFD with Cur, Pip or Cur + Pip had no effect on the rate of weight loss. Interestingly, the percentage of body fat loss during Phase 3 was significantly affected by supplementation with Cur + Pip. This observation indicates that when mice on a HFD are subjected to 20% CR, the initial loss of total body fat occurs with a minimal loss of lean body mass. The percent of body fat, which changed over 20 weeks, is better depicted as AUC in Fig. 3. Using AUC, we could perform statistical analysis and demonstrate the significant impact of Cur + Pip on modulation of total body fat. Supplementing HFD with Cur or Pip alone could not suppress adiposity beyond CR’s effect. As previously noted, plasma glucose and insulin levels were not affected by Cur or Pip alone or Cur + Pip beyond CR effect [8]. These results indicate that the impact of 20% CR on total body weight reduction of HFD-fed mice was high, and Cur + Pip supplementation was effective at further reducing of body fat.

One of the deleterious effects of elevated total body fat is the presence of low grade chronic inflammation, which can lead to metabolic syndrome and its associated diseases including cancer, diabetes, cardiovascular disease, inflammatory bowel disease, neurodegenerative disease, pancreatitis and psoriasis [4]. Therefore, we measured a battery of inflammatory cytokines in the plasma of the 4 CR groups treated with HFD with and without Cur (Groups 1–3 and 5). Daily intake of curcumin reduced several inflammatory cytokines in HFD-fed mice [5, 6, 9], we did not see a significant effect of Cur alone on plasma levels of inflammatory cytokines (Fig. 4). However, we found that CR mice fed HFD and supplemented with Cur + Pip had significantly lower IL-1β and KC/GRO concentrations in plasma compared to HFD ad libitum. In general, CR reduces expression of inflammatory cytokines [25]. Other inflammatory cytokines (IFN-γ, IL-10, IL-12 p70 and IL-6) were not affected by including CR, CUR and Pip in the HFD, which can be due to the presence of very low background levels in this study. On the other hand, Pip alone might be worked as antioxidant in Cur + Pip administrated group. To prove this, it would be needed for the confirmation of Pip concentration in the body. We analyzed Cur metabolites by HPLC-MS/MS to investigate whether decreased levels of IL-1β and KC/GRO depend on the alteration of Cur metabolism by Pip (Fig. 5). Analysis revealed that most of the Cur in plasma was conjugated as CurG. Other conjugates (CurS, CurDG, CurDS and CurGS) and Cur were found at extremely low levels in plasma. In general, almost of orally administrated Cur are discharged into feces. In the small intestine, Cur is subjected to conjugation reaction with UDP-glucuronosyltransferase or sulfotransferase by the second phase reaction (phase 2 metabolism) to provide CurG. Then, small amount of Cur and CurG are absorbed from the small intestine, transported to the liver via the portal vein, and undergoes a further conjugation reaction by the phase 2 metabolism. After that, it circulates in the blood and reaches various organs [18, 26]. These data support our previous observations that CurG is the major metabolite found in rodent plasma after administration of Cur [18]. We found no significant difference in CurG levels between groups supplemented with Cur alone or combined with Pip. This indicates that Pip did not alter Cur’s metabolism under the present experimental conditions.

It is reported that Cur conjugation (e.g., glucuronidation) occurs in the liver and intestines [26, 27]. Pip reportedly inhibits liver and intestinal glucuronidation activity possibly due to the presence of a conjugated double bond in the Pip molecule [28]. Shoba et al. performed a single oral dose study (2 g Cur/kg and 20 mg Pip/kg body weight) and found that Pip increased absorption and bioavailability of Cur by 200% in rats [7]. As far as we know, this report is the only one report that showed quantitative relationship between CUR and Pip intake. Compared to our study (9 mg/kg), they used higher amount of Pip (20 mg/kg). It needs to clarify the effective amount of Pip dose on Cur metabolism. Hlavačková et al. performed a daily administration study (100 mg Cur + 20 mg Pip/kg body weight for 6 weeks) and reported that Cur + Pip administration favorably impacted N-nitro-L-arginine-methylester-induced hypertension in rats [29]. In our study, the daily dose of Pip was approximately 9 mg/kg body weight [30]. Our study dose may be too low to increase Cur’s absorption and bioavailability substantially. Thus, we can deduce that regulation of telomere length and the decrease of cytokines (IL-1β and KC/GRO) have no connection with the potentially modifying effect of Pip on Cur metabolism. On the other hand, Pip is considered to have anti-inflammatory properties itself [31]. Hence, it is possible to deduce that decrease of these cytokines and fat loss would be enhanced by anti-oxidative activity of Pip and Cur. Also, further study will need to clarify the anti-inflammatory effects of Pip.

A study by Kanitkar et al. reported that daily Cur intake (7.5 mg/kg body weight) reduced IL-1β in the serum of STZ-induced diabetic mice [32]. In the present study, we found a negative correlation between plasma CurG and IL-1β (Table 1), which could possibly be attributed to the antioxidant activity of CurG. This outcome warrants future investigation.

Telomere length is associated with oxidative stress and aging [20]. CR and dietary bioactive antioxidants such as Cur and grape seed extract have been shown to be effective in protecting and maintaining telomere length [33]. Therefore, we sought to determine the impact of 20% CR in combination with the antioxidant activities of Cur + Pip on maintenance of liver telomere (Fig. 6). As a result, minor modulation of telomere length was observed. The possibility is that including CR in HFD-fed mice might have induced oxidative/lipid stress resulting in a shorter telomere [24]. However, Cur and Pip attenuated oxidative stress and affected telomere lengths to some extent because both Cur and Pip have anti oxidative activity. It might appear that stress of CR in HFD fed mice induces an oxidative stress condition which results in more shortening of the relative telomere length. Several researches showed sudden calorie restriction had a potential of oxidative stress. [34] In the present study, we mainly focused to diet. However, it has a potential of age-related changes in the metabolism [35]. Because, our study period undertaken for 53 weeks and it had a potential of metabolic alternation within the study. In the future, it needs to confirm the effect of aging on CUR and Pip with the obesity. Further studies are needed to clarify the impact of Cur + Pip on attenuating oxidative stress under CR and telomere length.

## Conclusions

We found that supplementing HFD of mice undergoing 20% CR with a combination of Cur + Pip may accelerate CR induced loss of total body fat. This combination also appears to be an effective intervention at suppressing HFD induced inflammation. In summary, combination of Cur and Pip appear to have the potential to enhance CR effects for the prevention of metabolic syndrome. Both of which are associated with good health status.

## Abbreviations

AUC: Area under the curve
CR: Caloric restriction
Cur + Pip: Curcumin and piperine
Cur: Curcumin
CurDG: Curcumin diglucuronide
CurDS: Curcumin disulfate
CurG: Curcumin glucuronide
CurGS: Curcumin glucuronide sulfate
CurS: Curcumin sulfate
DHCur: Dihydrocurcumin
DHCurG: Dihydrocurcumin glucuronide
DHCurS: Dihydrocurcumin sulfate
HFD: High-fat diet
HHCur: Hexahydrocurcumin
HHCurG: Hexahydrocurcumin glucuronide
HHCurS: Hexahydrocurcumin sulfate
HPLC-MS/MS: Chromatography with tandem mass spectrometry
IFN: Interferon
IL: Interleukin
KC/GRO: Keratinocyte chemoattractant / human growth-regulated oncogene chemokines
MRI: Magnetic resonance imaging
MRM: Multiple reaction monitoring
MSD: Meso scale discovery
OHCur: Octahydrocurcumin
OHCurG: Octahydrocurcumin glucuronide
OHurCS: Octahydrocurcumin sulfate
Pip: Piperine
T:S ratio: Ratio of telomere repeat to single copy gene
THCur: Tetrahydrocurcumin
THCurG: Tetrahydrocurcumin glucuronide
THCurS: Tetrahydrocurcumin sulfate
